# A Controlled Design of Aligned and Random Nanofibers for 3D Bi-functionalized Nerve Conduits Fabricated via a Novel Electrospinning Set-up

**DOI:** 10.1038/srep23761

**Published:** 2016-03-29

**Authors:** Jeong In Kim, Tae In Hwang, Ludwig Erik Aguilar, Chan Hee Park, Cheol Sang Kim

**Affiliations:** 1 Department of Bionanosystem Engineering, Graduate School, Chonbuk National University, Baekjaedae-ro, Dukjin-gu, Jeonju 561-756, Republic of Korea; 2 Department of Medical Practicing, Woori Convalescent Hospital, Andukwon-ro, Dukjin-gu, Jeonju 54914, Republic of Korea; 3 Division of Mechanical Design Engineering, College of Engineering, Graduate School, Chonbuk National University, Baekjaedae-ro, Dukjin-gu, Jeonju 561-756, Republic of Korea.

## Abstract

Scaffolds made of aligned nanofibers are favorable for nerve regeneration due to their superior nerve cell attachment and proliferation. However, it is challenging not only to produce a neat mat or a conduit form with aligned nanofibers but also to use these for surgical applications as a nerve guide conduit due to their insufficient mechanical strength. Furthermore, no studies have been reported on the fabrication of aligned nanofibers and randomly-oriented nanofibers on the same mat. In this study, we have successfully produced a mat with both aligned and randomly-oriented nanofibers by using a novel electrospinning set up. A new conduit with a highly-aligned electrospun mat is produced with this modified electrospinning method, and this proposed conduit with favorable features, such as selective permeability, hydrophilicity and nerve growth directional steering, were fabricated as nerve guide conduits (NGCs). The inner surface of the nerve conduit is covered with highly aligned electrospun nanofibers and is able to enhance the proliferation of neural cells. The central part of the tube is double-coated with randomly-oriented nanofibers over the aligned nanofibers, strengthening the weak mechanical strength of the aligned nanofibers.

The anatomical recovery of damaged peripheral nerves with their proper neurological functions after injury remains a significant clinical challenge^1^. Autologous nerve graft transplantation has been recommended as a form of first-line therapy for severe peripheral nerve injury^2^,^3^,^4^. However, autologous nerve graft transplantation has inevitable limitations, such as the possibility of permanent neural function loss of harvested donor nerves, limited choice of autologous grafts, and even a mismatch between the damaged nerves and an autologous graft can occasionally occur^5^,^6^,^7^. Due to the major limitations of the autologous graft ransplantation, this surgical method has been substituted with an artificial nerve guide conduit (NGC) that can address the limitations in autologous graft transplantation, especially those related to harvesting. An artificial nerve guide conduit (NGC) can also provide damaged nerves with favorable conditions as follows. First, the structure designed for the NGC has a selectivity that prevents the invasion of scar tissue into the NGC cavity through the NGC surface, but allows oxygen and nutrients for neuro regeneration to permeate through the NGC^1^. Second, the flexibility in the structural design to adjust the diameter and wall thickness and so on allows the damaged neurons to avoid nerve compression, which often causes post-operative complications including chronic inflammation, pain, etc^8^,^9^. Finally, the immunological inertness with the surrounding body tissue and bio-degradability of the NGC can also reduce post-operative complications and can improve the nerve restoration rate. The nanofiber-based scaffolds are usually fabricated using three different methods: phase separation, self-assembly, and electrospinning. Among those three nanofiber weaving methods, electrospinning is more widely used due to its superior adjustability in diameter, alignment, and signal encapsulation of the nanofibers^10^,^11^. Several studies have shown the excellence of nanofibrous scaffolds in terms of tissue regeneration and cell response^12^,^13^,^14^. In particular, nanofiber scaffolds with a high alignment exhibit remarkable advantages in terms of their mechanical properties and cell proliferation processes, such as cell orientation, migration, and differentiation. Highly-aligned nanofibers play a crucial role in neurite outgrowth of the neural cell when compared to randomly-distributed nanofibers^15^,^16^. Neurite outgrowth, cell migration and the extracellular matrix deposition of the neural cells on the aligned nanofibers tend to stretch in a parallel direction along the alignment of the nanofibers^17^,^18^,^19^.

In this study, we have developed a method to fabricate highly-aligned electrospun NGCs with selective permeability using various materials including poly lactic-co-glycolic acid (PLGA) and polyurethane (PU). The external side of the central part of the nerve conduit is coated with randomly-aligned nanofibers to increase its mechanical strength, and the internal side of the conduit is coated with growth factor-bearing aligned nanofibers for improved nerve regeneration^18^,^19^. COMSOL is used to simulate the distribution of the electric field of the modified electrospinning method to fabricate a highly-aligned nanofibrous mat. Different voltages between the needles and the collector are applied to investigate the behavior and the effect of the electric field while the strength and potential variation of the electric field between the needle and the cellophane tapes attached on copper wires are investigated. An electrospun, aligned nanofibrous matrix has a transparency that is different from that of randomly-aligned a nanofiber matrix, and this property plays a vital role in follow-up close observation during nerve reconstructive surgery^6^,^20^.

## Result and Discussion

### Fabrications of a mat in an angled U-shape

**Figure 1:**
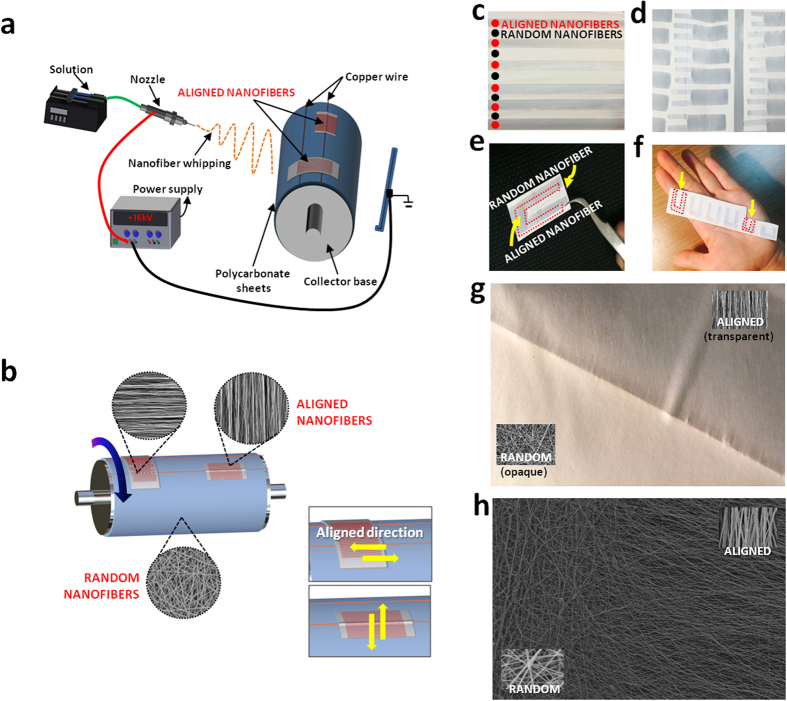
(**a**) Schematic illustration of fabrication for the controlled design of aligned and randomly oriented nanofibers (**b**) Schematic illustration of the direction of the nanofiber alignment influenced by the cellophane tape attachment (**c**) Macrograph of the mat with randomly oriented nanofibers with aligned nanofibers at regular intervals (**d**) Single mat with multiple angled U shape aligned nanofibrous mat (**e**) Single angled U shape aligned nanofibrous mat (**f**) Different sized angled U shape aligned nanofibrous mat (**g**) Macrograph of border of aligned and random nanofibrous mat (**h**) SEM image of border of aligned and random nanofibrous mat.

**Figure 2:**
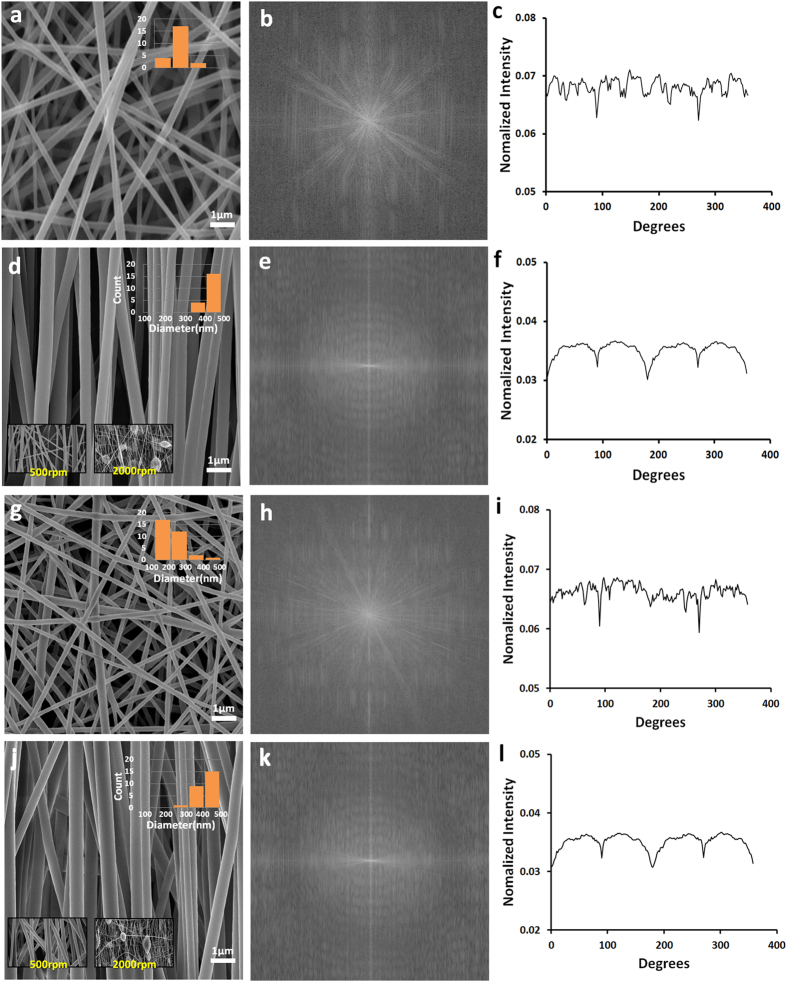
Morphological analysis of PU and PLGA nanofibrous mat with diameter graph (**a**) SEM image of randomly oriented PU nanofibers (**d**) SEM images of aligned PU nanofibers at a speed of 1000 rpm. inset; 500 rpm and 2000 rpm (**g**) SEM image of randomly oriented PLGA nanofibers (**j**) SEM images of aligned PLGA nanofibers at a speed of 1000 rpm, 500 rpm and 2000 rpm (**b,e,h,k**) FFT output images (**c,f,I,l**) Pixel intensity plots against the angle of acquisition for the aligned and random nanofibers.

Aligned and randomly oriented nanofibrous mats were prepared in one step using a modified electrospinning method as shown in Fig. 1a. The copper wires were set up horizontally on the collector and cellophane tapes were set over the fixed copper wires in the vertical and horizontal axis. Fig. 1a,b show that the electrospinning set up facilitates the fabrication of aligned and randomly-oriented nanofibers in one mat. In addition, the direction of the nanofiber alignment is influenced by the way the cellophane tape is attached on to the copper wires. A horizontal cellophane tape attachment yields a vertical nanofiber alignment and a vertical cellophane tape yields a horizontal nanofiber alignment (Fig. 1b). In the spinning process of the aligned nanofibers detailed above, a rotation speed between 800–1300 rpm of the collector base shows no particular importance, but the collected nanofibers tend to snap and deviate from alignment if the rotating speed is too quick or too slow. The optimal rotation speed for the collector was found to be 1000 rpm. Even though aligned nanofibers can be produced, even when the collector base stands still, the nanofibers exhibit a higher alignment when the collector rotates at a proper speed. This implicates that the modification can easily be made to any kind of electrospinning machine set-up with just partial modification on the collector aspect. One crucial aspect of this set-up is that it can fabricate aligned and random oriented nanofibers via one step process making it the only known study to do so (Fig. 1c,d). Also one of the advantages of this method is the controllability of the design aspect in which we can decide where to place the aligned and random nanofibers on to the finished mat and one example is shown in Fig. 1c. The NGC is made by rolling up the highly-aligned nanofibrous mats fabricated in an angled U-shape, as seen in Fig. 1d–f. The electrospinning set up shown in Fig. 1d can produce a single mat with multiple angled U-shaped nanofibrous mats with a diameter of 1 mm to 7 mm in a mat with just one electrospinning session. This electrospinning method has the capability to produce more than fifty nerve guide conduits at once, which shows its mass production capability. Moreover, the electrospinnig set-up can fabricate different sized angled U shape mat (Fig. 1f). Fabrication at a speed of 1000 rpm for the rotating collector shows the best alignment as shown in Fig. 2d,j. The nanofibers collected at high speed (1500–2000 rpm) tended to be disconnected, beaded and too thin as shown in the inset of Fig. 2d,j because solvent evaporation is outpaced by the fabricating speed on the cellophane tape at that higher speed. Moreover, high rotation speed could generate more air turbulence and wind which might scatter the nanofibers during electrospinning. As such, the nanofibers could break before they reach the collector, resulting in overall low mechanical strength. However, when the nanofibers were gathered at lower speed than 500 rpm, alignment of the nanofibers was usually not good. Because when the electrospinning jet hit the collector surface at lower speed than 500 rpm, the nanofibers could not collected on the rotating collector well coiled in the direction of rotation (Fig. 2d,j).

### Fiber alignment analysis via FFT and Image J

Fast Fourier Transfer (FFT) approach is applied to measure the alignment rates of the nanofibers, and ImageJ, image analysis software was used to analyze the FFT results^21^,^22^. The FFT analysis of the SEM images of the nanofiber samples was used to characterize the anisotropy of the scaffolds to digitize the alignment level of the nanofibers. Patterned, grayscale pixels are distributed in the output image of the FFT analysis to reflect the degree of fiber alignment of the original data image. A representative FFT analysis of randomly-oriented nanofibrous mats and aligned nanofibrous mats are presented in Fig. 2. A significant difference appears between the FFT images of the aligned nanofibers and randomly-oriented nanofibers were noted. The representative FFT of the original data image of the random fibers (Fig. 2b,h) generates an output image with symmetrically and circularly distributed pixels. On the other hand, the FFT data of the image with aligned fibers results in an output image with non-randomly and elliptically distributed pixels (Fig. 2e,k). The pixel intensities were plotted between 0 to 360°, and the degree of alignment in the FFT data reflected the shape and height of the peak shown on the plot. The higher the intensity and fewer occurrences of peaks on the plot indicate that the alignment of the nanofibers is highly ordered (Fig. 2c,f,i,l).

### Macro and Micro Morphology of the NGCs

**Figure 3:**
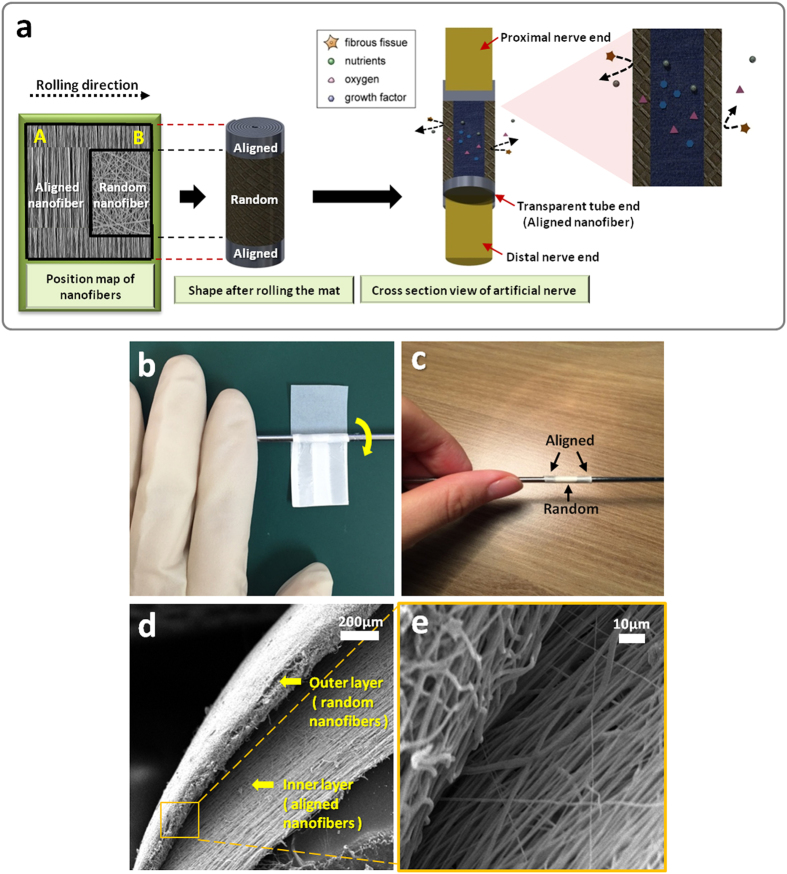
(**a**) Schematic 3D illustration of the nerve guide conduit (the position map of nanofiber, shape after rolling the mat and cross section view of the conduit is shown in this figure) (**b,c**) SEM images of the cross section view of the nerve tube (**d**) A digital photo of rolling the angled U shape mat on a rod (**e**) A digital photo of the nerve guide conduit after rolling.

Multiple angled U shaped patterns were made on a single mat using the modified electrospinning method. Each single angled U-shaped pattern was used as the final layout for the NGC; the schematic is shown in Fig. 1d,e. In order to create an NGC with an aligned nanofiber interior, we rolled the mat from part A to part B, as seen on Fig. 3a. The actual procedure of NGC formation can be seen on Fig. 3b,c. This would ensure that the interior part will have the aligned nanofibers and the exterior part of the NGC will have the random nanofiber orientation. The NGC has three main parts, the transparent proximal and distal portion, Opaque exterior medial portion, and an Aligned nanofiber interior. The aligned nanofibers can be seen on the proximal and distal end of the NGC and the rationale behind this is to ensure that the suturability of the NGC is improved by making the sutures visible to the surgeon^23^. The medial part of the NGC is made up of randomly oriented nanofibers, this is to increase the mechanical properties of the NGC and also to reduce the risk of fibrous tissue infiltration inside the nerve conduit. And lastly we designed the NGC to have an aligned nanofiber interior, so that the nerve cells can proliferate in line with the orientation of the nanofibers. Moreover, the conduit has nano to micro sized pores (50 nm-5 μm) that can permeate oxygen, nutrients, neurotrophic factors such as growth factors, and prevent the infiltration of fibrous tissues^1^ (Fig. 3a). The micro morphology of the PU and PLGA NGC samples were investigated using SEM. Figure 3d,e are the cross section of the central part of the tube that show the inner layer of the nerve conduit coated with aligned oriented PU nanofibers and an outer layer with randomly-oriented PU nanofibers. The average fiber diameter of the nanofibers on all samples can be seen on the inset of Fig. 2a,d,g,j. The average nanofiber diameter varies whether the nanofibers were aligned or randomly oriented. The aligned nanofibers shown a thicker nanofiber diameter and the random nanofibers has thinner nanofiber diameter in respect to the aligned ones. The reason behind this trend could be the difference on the electrical field that was generated by the copper wire and cellophane tapes. On the aligned nanofibers the electric field was constant at 0 V/m creating an increased bending stability and shorter path length for the polymer solution creating thicker nanofiber diameter.

### Characterization of scaffolds

#### Transparency and Porosity

**Figure 4:**
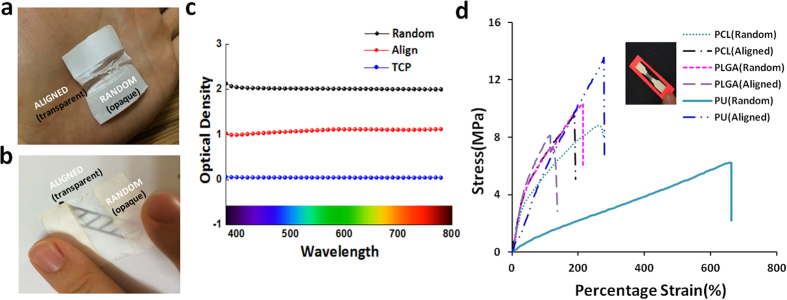
Transparency of nanofibers (**a**) A mat with aligned and randomly oriented nanofibrous mat (**b**) Macrograph of the mats with pattern for identifying the transparency of an aligned nanofibrous mat (**c**) Transparency results using a spectrum analysis method at the wavelength range of 380 nm–780 nm (**d**) Mechanical strength of PCL, PLGA and PU mat fabricated with aligned nanofiber and random nanofiber.

The transparencies of the scaffolds were carried out using spectrum analysis in the wavelength range from 380 nm to 780 nm, and Fig. 4c shows the aligned-oriented nanofibrous mat with a transparency that is twice higher than that of the randomly-oriented nanofibrous mat. Also, the characters written on a paper are observed to be readable through the aligned nanofibrous mat with the unaided eye (Fig. 4a,b). The transparency of the NGC’s proximal and distal parts has shown to have an advantage in allowing for direct observation to the incised nerve endings during surgery^23^,^24^. The porosity of the mats was characterized using the MicroActive AutoPore V 9600, and the porosity values of the aligned and randomly oriented PU nanofibrous mat that was obtained is given in Table 1. The MicroActive AutoPore V 9600 can be used for a mercury porosimetry analysis technique to obtain the porosity values from mercury intrusion into pores of the object under strictly-controlled pressures and conditions. The total intrusion volume of mercury for the aligned nanofibrous mat is larger than that for the randomly-oriented nanofibrous mat (Table 1). The greater value of the aligned-oriented nanofibrous mat implies that the mat allows for more mercury intrusion, and the volume of this mat is larger than the volume of the pores in the randomly-oriented nanofibrous mat. The average diameter of the pores in the aligned nanofibrous mat is three times larger than that in the randomly-oriented mat, and the porosity of the aligned nanofibrous scaffolds is greater. This is expected to result in less contact points between the nanofibers on the aligned nanofibrous mat than on the randomly oriented-nanofibrous mat.

#### Mechanical testing

A universal testing machine (AG-5000G, Shimadzu, Japan) was used to carry out the tensile properties of the matrices with woven, aligned PU and PLGA nanofibers at room condition. The testing samples were dog-bone shaped with a gauge length of 40 mm and a width of 10 mm, and tested at a speed of 5 mm/min. The sample seen in the inset of Fig. 4d consists of a 1 cm wide aligned nanofibrous middle part and the rest of randomly-aligned nanofibers. The thickness of the aligned nanofibrous mat ranges from 0.028 mm to 0.039 mm after 8 hours of electrospinning while the randomly-oriented nanofibrous mats ranges from 0.05 mm to 0.095 mm in thickness. The aligned nanofibrous mats also have a weaker mechanical strength relative to the randomly-oriented nanofibrous mat. As shown in Fig. 4d, a lower mechanical strength was observed in the aligned nanofibrous structures regardless of the type of polymer due to the fewer contact points for the aligned nanofibers.

#### Wettability

Cell adhesion to the biocompatible material, which is an essential prerequisite for healthy cell growth, cell migration and proper cell differentiation, is greatly affected by the physical surface properties and the chemical composition of the scaffolds. Since the cell adhesion to the scaffolds is mandatory for cell regeneration, the contact angle measurements is an important assessment to evaluate the cell behavior on the scaffolds. The wettability was influenced by various factors, such as the mat homogeneity, the type of fluid, and the surface tension measured according to the contact angle. As shown in Table 1, the contact angles between the substrate and the scaffolds were measured at 3, 6 and 9 seconds in this experiment. The hydrophilicity of nanofibrous mats could be clearly observed at a time interval of 3 seconds. Time dependency of wettability of scaffolds could influence the initial stage of cell attachment and proliferation after implantation of the scaffolds, this is due to the likelihood that cells can adhere faster on a substratum where it can readily absorb the cell adhesion molecules (CAM) produced by the nerve cells . The contact angle values of the randomly oriented and aligned nanofibrous mat was measured, and the average angles were 128.3° and 120.2° respectively. The increase in wettability can be attributed to the increase in porosity and the acicular shape of the pores of the aligned nanofibers^25^,^26^. This result shows that aligned nano pattern surface can be advantageous for fabricating polymeric biomedical scaffolds with strong wettability properties.

#### Electric field analysis

**Figure 5:**
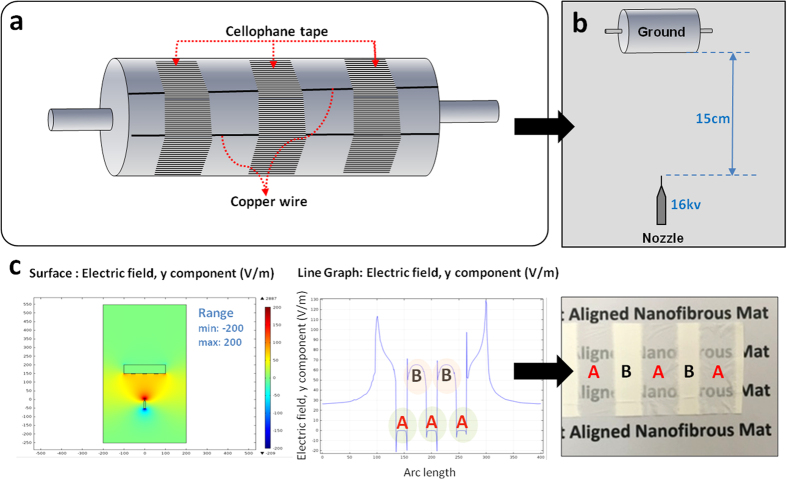
(**a,b**) The set up for fabrication of designed mat with aligned and random nanofibers (**c**) Results of electric field distribution simulation (Top view): surface and variation of electric field.

The presence of the electric charges makes the electric force and the electric field to become the area where an electric force exists. This is a significant point to account for the interaction between two sites of a different electric charge, and if there are more sites that affect the electric field in the system, there is potential to change the electric charge^27^,^28^. It is possible to control the orientation of the aligned or the randomly-oriented nanofibers during the modified electrospinning by adjusting the electric field. The nanofibers that are collected on the cellophane tape were aligned in parallel due to the adjustment of the electric field between the spinning needle and the collector caused by the copper line and the cellophane. COMSOL® Multiphysics software was used to guide the actual manufacturing method to analyze the effects of the electric field on the nanofiber alignment and configuration. An electric field analysis was carried out using the COMSOL (ver. 4.3) add-on AC/DC module on a computer running the Windows Vista operating system. The simulations were performed using the actual configuration of the electrospinning set-up, as shown in Fig. 5a. COMSOL® enables the analysis of the electric field simulations from the top view, and the representations of the surface of the electric field are also presented in Fig. 5b. Furthermore, Fig. 5c represents the distribution of the electric field on the surface whereby the direction is denoted using a scale bar (max. 200 V/m, min. −200 V/m). The effect of the direction of the cellophane tape on the intensity of the electric-field is shown in Fig. 5c. The electricfield value (V/m) for the aligned nanofibers was constant at 0 V/m as presented by letter A, and for the random nanofibers, fluctuations can be seen in the electric field values as presented by letter B on Fig. 5c. The fluctuations create random orientation due to the increased whipping, instability and interaction between the polymeric jets. According to the horizontal line graph, the direction of the electric-field direction is shown to repeatedly change from the (+) direction to the (−) direction. According to the overall results, the electric field was evenly-distributed and pointed change, adhering to the required conditions to produce aligned electrospun nanofibers. The data that we learn from the simulation phase of the experiment was applied on the modification of the eletrospinning set-up, in order to produce the aligned and random oriented nanofibers.

#### Cell morphology and viability

**Figure 6:**
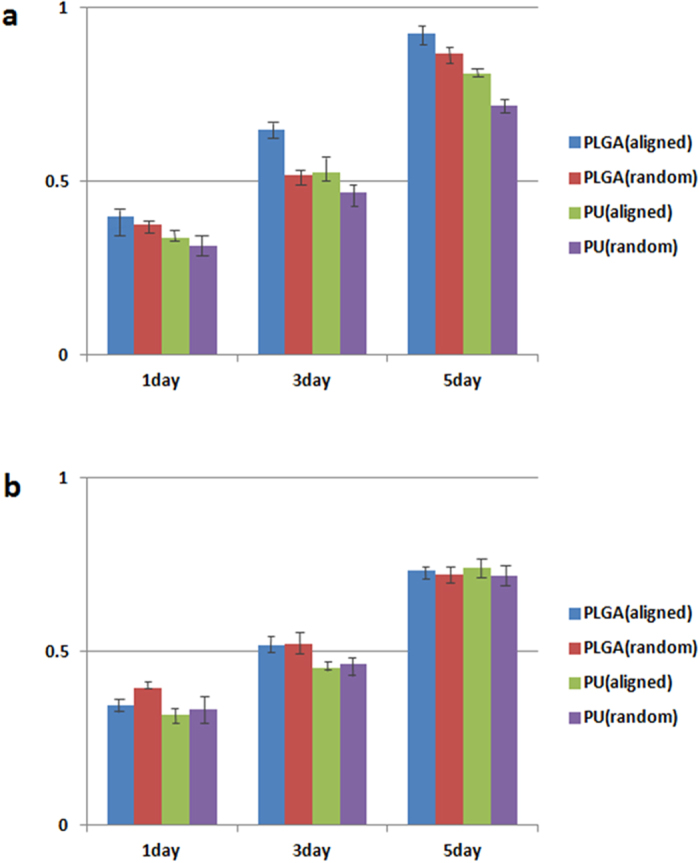
(**a**) CCK-8 assay result of PC12 cells on PLGA and PU nanofibrous mats after 1, 3 and 5 days of cell culture (**b**) CCK-8 assay result of S42 cells on PLGA and PU nanofibrous mats after 1, 3 and 5 days of cell culture. The data is reported as the mean ± standard deviation (n = 5, p < 0.05).

**Figure 7:**
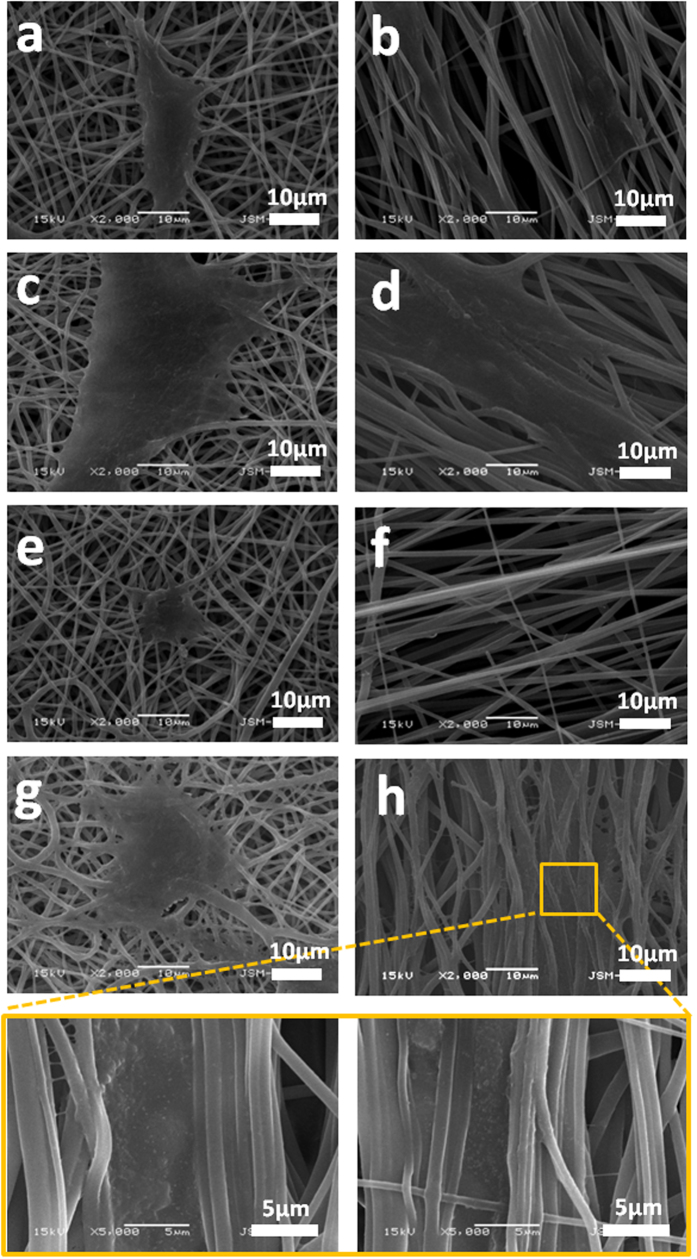
(**a–d**) Morphology of PC12 cells on different mats after 1 day and 5 days cell culture **(e–h)** Morphology of S42 cells on different mats after 1 day and 5 days cell culture.

**Figure 8:**
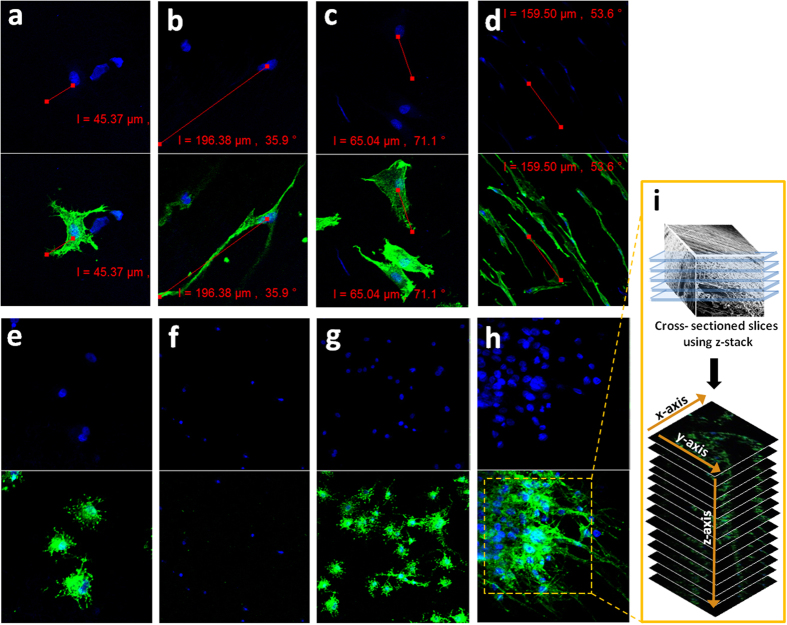
(**a,b**) Confocal microscopy images of PC12 cells attached after 1 day of culture on a randomly oriented and aligned nanofibrous PLGA scaffold (**c,d**) Confocal microscopy images of PC12 cells attached after 5 day of culture on a random and aligned nanofibrous PLGA scaffold (**e,f**) Confocal microscopy images of S42 cells attached after 1 day of culture on a random and aligned nanofibrous PLGA scaffold (**g,h**) Confocal microscopy images of S42 cells attached after 5 day of culture on a random and aligned nanofibrous PLGA scaffold. Actin Green 488 (green) was applied for actin fillament and DAPI (blue) for staining nuclei (**i**) Z stack from a PLGA scaffold with aligned nanofibers in which S42 cells. Images were collected at 0.37 μm intervals using the 488 laser.

The surface matrix of the electrospun scaffolds can provide a supportive condition for adhesion and proliferation of the cells due to the similarities in the physical properties and neural tissue structure^29^. In this study, PC12 cells and S42 cells were seeded on PLGA and PU nanofibrous mats with aligned and random orientations. Each scaffold with cells was cultured for 1, 3 and 5 days and *in vitro* biocompatibility evaluations were done by using CCK-8 assay, SEM and confocal laser scanning microscopy. It has been ascertained from the CCK-8 assay result, as shown in Fig. 6, that after days 1, 3 and 5, the proliferation of PC12 cells on the PLGA samples were higher compared to PU samples. However, a higher proliferation was noted on both polymer samples on the aligned nanofiber orientation. The SEM images in Fig. 7 show that the cell growth on the aligned nanofibrous mat tends to be vigorous and faster compared to the cell growth on the randomly-oriented mat. From the cell morphologies on the different alignments, the cells appear to be well-attached to the surface of the aligned nanofibers compared to the randomly-oriented nanofibers. It is well known that the surface hydrophilicity of the scaffolds can cause a higher initial cell adhesion to the surface^30^,^31^,^32^,^33^. Figure 7a–d show an increase in the PC12 cells proliferation on the aligned nanofibrous scaffolds compared to the cell proliferation of the randomly-oriented nanofibrous mats on the first day after cell seeding due to the higher hydrophilicity of its surface. However, even though the wettability of the aligned nanofiber is greater than the randomly oriented nanofibers and should potentially increase the initial proliferation rate of the S42 cells, we observed the opposite. This is possibly due to the small size of the S42 cells compared to the aligned nanofiber gaps. The cells finds it difficult to proliferate due to the limited substratum space. But eventually S42 cells adhered and proliferated after day 5 as shown in Fig. 7f,h. After 5days, there appears to be no significant difference in the cell numbers between both the electrospun scaffolds, implying that the proliferation of cells on aligned nanofibrous scaffold after the cell adhesion phase outpaces that on randomly-oriented nanofibrous scaffolds. An exception to this case was the S42 cells seeded on aligned nanofibers, it appears to have relatively low cell proliferation rates compared to the viabilities of the cells on the random nanofibrous mat at day 1 (Fig. 7e,f). Confocal laser scanning images also show the cell proliferation behavior and the fiber-cell interaction according to the alignment rate of the nanofibers over the scaffolds. As expected, the cell adhesion on the surface and cell proliferation rate is higher for the aligned nanofibers than the randomly-oriented nanofibers as seen in the confocal miscoscopy image (Fig. 8g,h). In Fig. 8a–d, the length of the actins of the PC12 cells cultured on aligned oriented nanofibrous matrix, which were dyed with actin green, were observed to be almost three times longer. Also, the cells on the aligned nanofibrous matrix tend to be spindle-shaped and grew longer and become more elongated and developed along with the aligned nanofibers. However, in the case of the S42 cell growth, as the CCK-8 assay results imply, the actin filament length of the S42 cell on the aligned nanofibers didn’t express enough on day 1 because the S42 cell’s limited substratum space (Fig 8e,f). Nevertheless, an occurrence of the proper cell proliferation and cell adhesion to the nanofibers inside of the gaps of the nanofibers rather than the surface of the nanofibrous matrix was observed in the S42 confocal image on day 5 (Fig 8g,h). The size of the S42 cells is quite smaller compared than that of ordinary cells, and thereby, their cell adhesion to the aligned nanofibers occurs after falling through the gaps between the nanofibers. This being so, PC12 cells grows well on the aligned nanofibrous mat compared to randomly-aligned nanofibrous mat from day 1, but the S42 cells took 4 more days to get on track. To further validate whether the S42 cells did penetrate the gaps in between the nanofibers, we did a Z stack confocal imaging after day 5, each cross sectioned image has a thickness of 0.37 μm. The results can be seen in Fig. 8i and the images were collected at 0.37 μm intervals using the 488 laser. As shown on the image, the depth of the S42 cell growth can be observed from the top image up to the bottom image with an overall depth of 28.85 μm, indicating that the cells did penetrate the scaffold.

Consequently, scaffolds with aligned-oriented nanofibers can facilitate and accelerate nerve regeneration due to the nature of the nerve cells that tend to flourish (grow well) along with the aligned nanofibers. The advanced hydrophilicity and the adhesive property of the aligned nanofibrous matrix might play other key roles for better cell proliferation. These results indicate a superiority of the aligned nanofibrous scaffold to randomly-oriented nanofibrous scaffolds as a substrate for tissue engineering, especially regarding cell proliferation and guidance. Therefore, aligned-oriented nanofibrous scaffolds can be a candidate for extensive use in nerve tissue engineering.

## Conclusion

In summary, we have successfully invented a modification of the electrospinning process in order to fabricate highly-aligned nanofibers and randomly-oriented nanofibers on a single mat via a single step process. In addition, the electrospun aligned and random oriented nanofibers can be easily rolled into an angled U-shape to form an NGC that can be used as a practical way to assist nerve regeneration. In this work, the fabricated NGCs show high transparency and could allow the incised nerve fibers in the conduits to be seen directly by the naked eyes during implantation. This improvement in transparency of both the edges of the tube with aligned nanofibrous mat could potentially facilitate the ease of nerve reconstructive surgery. The central exterior portion of the tube where the random nanofiber is located could prevent scar tissue invasion while the inner coating aligned nanofibers can help vital active nerve regeneration. The fabricated scaffold with aligned nanofibers has superb hydrophilic properties that can facilitate in cell proliferation and cell growth guidance. The aligned nanofibers acted as a guide for neural cells and were able to achieve a higher cell proliferation and migration compared to randomly oriented nanofibers. This processing method can be a promising candidate for fabricating scaffolds for nerve tissue engineering.

## Materials and Methods

### Materials

Co-polymers of poly (lactic- co -glycolic acid) (PLGA) and polyurethane (PU) were obtained from Sigma Aldrich, Korea to produce the aligned oriented nanofibrous mat. Tetrahydrofuran (THF) and N, N dimethylformamide (DMF) were obtained from Showa (Japan) and were prepared for use as a solvent. Mouse Schwann cells (S42) and Rat pheochromocytoma cells (PC12) were obtained from the Cell Line Bank. Culture reagents including Dulbecco’s modified Eagle’s medium (DMEM), antibiotic solutions and fetal bovine serum (FBS) were purchased from Sigma. Mouse nerve growth factor (NGF) was obtained from Sunlong Biotech (Korea).

### Fabrication of NGCs

In this experiment, the 10 wt% concentration of PLGA and PU solutions were dissolved with mixed solvent containing 1:1(by weight percent) of THF and DMF with continuous magnetic stirring. Random, aligned electrospun nanofibrous scaffolds made of co-polymer of poly (lactic- co -glycolic acid) (PLGA) and polyurethane (PU) were fabricated using the electrospinning set-up shown in Fig. 1a. Electrospinning was carried out in room conditions with a high-voltage power supply of 16 kV and needle tip-to-collector distance of 15 cm. The solution feed rate was set to 1 ml/h. The modified electrospinning method to produce aligned fibers consists of a conductive collector with copper wires and cellophane tapes on the wires (Fig. 1a). The copper wires were horizontally stuck onto the rotating collector and the cellophane tapes were attached horizontally and vertically on the copper wires. Finally, the nanofiber mats were vacuum-dried in an oven at 30 °C for 24 h to remove the residual solvent, and the sample was then used for further analysis.

### Characterizations of nanocomposite scaffolds

The fiber morphology and the cell adhesion of the electrospun PU and PLGA nanofibers were observed using field-emission scanning electron microscopy (FE-SEM, Hitachi S-7400, Hitachi, Japan) and scanning electron microscopy (SEM, Hitachi S-7400, Hitachi, Japan). Samples collected on from the rotating collector with a polyethylene sheet were prepared for each experiment. The mechanical properties of the mats in the longitudinal direction were measured by using a universal testing machine (AG-5000G, Shimadzu, Japan) at room temperature. To determine the wettability of the electrospun nanofibrous mats, the water contact angle was measured with deionized water using a contact angle meter (GBX, Digidrop, France). All experiments were conducted in room conditions and were performed at different time intervals of 3, 6 and 9 s for more than 5 times per sample. The permeability and the mechanical properties of the nanofibrous scaffolds were determined according to their porosity, and the porosity of the PU fibrous mats was measured using MicroActive AutoPore V 9600 (Micromeritics Instrument, Korea). The measurements of the film scaffold transparency was carried out using a spectrum analysis method by SYNERGY Mx spectrophotometer (BioTek^R^, USA) in a wavelength range from 380 nm to 780 nm. The FFT method was used to evaluate the fiber alignment of the fabricated mats. The alignment of the fibers in the images was analyzed using the ImageJ software. During the FFT analysis, the alignment degree of the data image was reported by the FFT peak shapes and the height. The FFT utilization has the point to systematically evaluate the correlation and causality between the alignment degree and the other structural and material properties of the scaffold by setting up the relative numerical values of the alignment of the electrospun fibers^34^,^35^.

### Cell culture

PC-12 cells (Cell Line Bank, Korea) were cultured in DMEM (Gibco) medium supplemented with 1% penicillin/streptomycin, 5% horse serum(Gibco) and 5% calf serum(Gibco) and 1% NGF (Sunlong Biotech, Korea) at 37 °C with 5% CO_2_ in an incubator, and S42 cells (Cell Line Bank, Korea) were cultured in DMEM medium including 10% FBS(Sigma), 1% penicillin/streptomycin. The electrospun nanofibrous scaffolds were cut to their proper size (1.2 cm × 1.2 cm) to cover the cell culture plate (SPL life science, Korea). After sterilization under UV radiation for 12 hours, the scaffolds were washed 3 times with phosphate buffered saline solution (PBS), and cells with density of 2** × **10^4^ cells per well were seeded on the electrospun mats and were cultured in DMEM medium in a humidified atmosphere with 5% CO_2_ at 37 °C. The samples were then transferred to individual 48-well tissue culture plates. The adhesion and proliferation of the cells on the nanofibrous scaffolds was measured using the cell counting kit-8 after 1, 3 and 5 days of cell culture. At desired points in time, the CCK-8 solution was added to each well and was incubated for 2 hours in 37 °C incubator with 5% CO_2_. The measurement of the absorbance was checked at 450 nm using a microplate reader (Tecan, Austria). A morphological study was carried out for the *in vitro* cultured PC12 cells and S42 cells on aligned and randomly-oriented electrospun nanofibrous scaffolds for 1 day, 3 days and 5 days. First, the cells were grown on scaffolds for 1, 3 and 5 days and were washed three times with PBS to remove the non-adherent cells. The attached cells were fixed with 2.5% glutaraldehyde solution, and were then dehydrated in ethanol solution through a graded series of alcohol (50, 60, 70, 80, 90, and 100%) and were dried for 12 hours in a clean bench. The morphology was then examined by using SEM and FE-SEM.

### Confocal laser scanning microscopy

The PC12 cells and S42 cells were seeded on aligned and randomly-oriented electrospun nanofibrous scaffolds (PLGA, PU) with a density of 2** × **10^4^ cells per well and were incubated for 1, 3 and 5 days. The media with the cultured cells was aspirated from wells, and the attached cells were rinsed with PBS solution. The cells were then fixed with 4% paraformaldehyde (PFA) solution for 10 min at room temperature and were permeabilized in 0.2% triton X-100 in PBS for 2 min. After blocking with 1% HSA/PBS dilution for 30 min, the nanofibrous scaffolds were stained with Actin-green488 solution and DAPI in each of the 24 wells and were incubated in the dark for cell nuclei and actin filament staining. Cells fluorescence was then observed using a fluorescence microscope.

## Additional Information

**How to cite this article**: Kim, J. I. *et al.* A Controlled Design of Aligned and Random Nanofibers for 3D Bi-functionalized Nerve Conduits Fabricated via a Novel Electrospinning Set-up. *Sci. Rep.* **6**, 23761; doi: 10.1038/srep23761 (2016).

## Figures and Tables

**Table 1:** Total intrusion volume at 32,994.69 psia, average pore diameter and porosity and Contact angle measurements of random and aligned nanofibers.

	Total intrusion volume [at 32,994.69 psia]	Average pore diameter [4V/A]	Porosity	Contact angle		
				3s	6s	9s
Aligned	1.34 mL/g	1.56 μm	27.86%	120.8°	120.2°	119.8°
Random	1.11 mL/g	0.54 μm	24.64%	128.9°	128.3°	127.7°
